# Improved Heuristics for Minimum-Flip Supertree Construction

**Published:** 2007-02-28

**Authors:** Duhong Chen, Oliver Eulenstein, David Fernández-Baca, J. Gordon Burleigh

**Affiliations:** 1 Department of Computer Science, Iowa State University, Ames, IA 50011, U.S.A; 2 Section of Evolution and Ecology, University of California, Davis, CA 95616, U.S.A

**Keywords:** Supertree, phylogenetic trees, matrix representation with flipping, matrix representation with parsimony, tree search heuristics

## Abstract

The utility of the matrix representation with flipping (MRF) supertree method has been limited by the speed of its heuristic algorithms. We describe a new heuristic algorithm for MRF supertree construction that improves upon the speed of the previous heuristic by a factor of *n* (the number of taxa in the supertree). This new heuristic makes MRF tractable for large-scale supertree analyses and allows the first comparisons of MRF with other supertree methods using large empirical data sets. Analyses of three published supertree data sets with between 267 to 571 taxa indicate that MRF supertrees are equally or more similar to the input trees on average than matrix representation with parsimony (MRP) and modified min-cut supertrees. The results also show that large differences may exist between MRF and MRP supertrees and demonstrate that the MRF supertree method is a practical and potentially more accurate alternative to the nearly ubiquitous MRP supertree method.

## Introduction

There is increasing interest in supertree methods for phylogenetics (see Bininda-Emonds et al. 2002; Bininda-Emonds, 2004). Supertree methods combine phylogenetic trees with incomplete taxonomic overlap into a comprehensive phylogeny that incorporates all taxa from the input trees. Since the ultimate aim of many supertree analyses is to build large phylogenies, an effective supertree method must be fast as well as accurate. Therefore, the development and implementation of fast algorithms is a critically important part of establishing useful supertree methods.

The most popular supertree method by far is matrix representation with parsimony (MRP; see Bininda-Emonds, 2004). MRP performs a maximum parsimony analysis on a binary matrix representation of the set of input trees (Baum, 1992; Ragan, 1992; Baum and Ragan, 2004). Therefore, MRP analyses can use fast maximum parsimony heuristics (e.g. Nixon, 1999; Goloboff, 2000) and popular phylogenetics programs that implement maximum parsimony like PAUP* (Swofford, 2002) or TNT (Goloboff, 2000). Still, MRP has been criticised for its performance and properties (e.g. Purvis, 1995; Pisani and Wilkinson, 2002; Gatesy and Springer, 2004; Goloboff, 2005; Wilkinson et al. 2005). For example, MRP may have a size bias, in which the size of the input trees affects how MRP resolves conflicts (Purvis, 1995). There is also evidence that MRP may have a shape bias, in which input tree shape affects the resulting supertree (Wilkinson et al. 2005). Furthermore, the validity of using parsimony on a matrix representation of input trees has been questioned (Slowinski and Page, 1999; Eulenstein et al. 2004; Gatesy and Springer, 2004). Thus, there is a need to investigate alternate supertree methods.

The matrix representation with flipping (MRF, or minimum flip) supertree method, like MRP, uses a matrix representation of the input trees (Chen et al. 2003; Chen et al. 2004; Burleigh et al. 2004; Eulenstein et al. 2004). While MRP seeks trees that minimize the parsimony score of the matrix representation of input trees, MRF seeks the minimum number of flips, character changes from 0 to 1 or 1 to 0, that will make the matrix representation of input trees consistent with a phylogenetic tree (see Chen et al. 2003; Burleigh et al. 2004; Eulenstein et al. 2004). The resulting phylogenetic trees are MRF supertrees. Like the parsimony problem, the minimum flip problem is NP-hard (Chen et al. 2006), and therefore, estimating an MRF supertree requires heuristic algorithms when the input trees contain more than approximately 20 taxa. Simulation experiments (Eulenstein et al. 2004; Piaggio-Talice et al. 2004) indicate that MRF supertrees retain more of the relationships from the input trees than MinCut (Semple and Steel, 2000), Modified MinCut (MMC; Page, 2002), and quartet supertree methods (Piaggio-Talice et al. 2004). Also, MRF supertrees retain relationships from the input trees at least as well as MRP supertrees, with MRF appearing to slightly outperform MRP as the taxon overlap among input trees decreases (Chen et al. 2003; Burleigh et al. 2004; Eulenstein et al. 2004). Though these results suggest that MRF is a promising supertree method, the characteristics of the simulated data sets likely differ greatly from those of empirical data sets. Furthermore, the first MRF heuristics were slow (Eulenstein et al. 2004; Goloboff, 2005), and consequently the performance of the MRF supertree method on large empirical data sets has been largely unexamined.

We describe improvements to existing MRF heuristics that increase the speed of the heuristics by a factor of *n*, where *n* is the total number of taxa represented in the input trees. These improvements make it feasible to estimate MRF supertrees for large data sets. Furthermore, they allow the first comparisons of the performance of MRF with other supertree methods using empirical data sets containing many more taxa than were in the simulated data sets. The results of these analyses demonstrate that MRF may perform better than MRP or MMC supertree methods. The analyses also demonstrate notable differences between results of MRF and MRP supertrees that were not observed in small simulation studies.

## Definitions

Let *S* = {*s*_1_, …, *s_n_*} denote a set of *n* taxa and ℒ(*T*) denote the leaf set of a rooted tree *T*.

A *directed phylogenetic tree*, or *phylogeny* for short, over set *S* is a rooted binary tree *T* such that every internal node of *T* has two children and ℒ (*T*) = *S*. (The assumption that the phylogenies are binary is made only for simplicity and can easily be dropped.) Let *v* be a node of a phylogeny *T*. Then, *T_v_* denotes the subtree rooted at *v*, and *T* − *T_v_* denotes the tree *T* with the subtree *T_v_* removed. The set ℒ (*T_v_*) is the *cluster* of *T* at *v*.

A *profile* is a multiset τ of phylogenies. The elements of τ are called *input trees*. A *supertree* for a profile τ is a phylogeny *T* such that ℒ(*T*) = ∪*_t_*_∈τ_ℒ (*t*).

A *character matrix* for *S* is an *n* × *m* matrix *M* =[*a_ij_*] over {0, 1, ?}, whose *i*-th row corresponds to taxon *s_i_*. The *j*-th column of *M* is called *character j*. The set of all *s_i_* such that *a_ij_* = 1 is the 1*-set* of character *j* and is denoted by *O_j_*; the set of all *s_i_* such that *a_ij_* = 0 is the 0*-set* of character *j* and is denoted by *Z_j_*.

Let τ be a profile such that ∪*_t_*_∈τ_ℒ (*t*) = *S*. For our study, we define a *matrix representation* of τ as a character matrix *M* for *S* obtained as follows. For each tree *t* ∈ τ and each cluster *X* in *t*, create a column of *M* whose *i-*th entry is 1 *if s_i_* ∈ ℒ *X*, 0 *if s_i_* ∈ ℒ(*t*) − *X, and* ? if *s_i_* ∉ ℒ (*t*).

The matrix representation of trees is the basis for MRP (Baum, 1992; Ragan, 1992; Purvis, 1995) and MRF (Chen et al. 2003; Burleigh et al. 2004; Eulenstein et al. 2004) supertree methods. We note that there are numerous different matrix representations of trees (e.g. Farris et al. 1970; Purvis, 1995; Wilkinson et al. 2004). In this paper, we use a standard binary matrix representation (Farris et al. 1970; Baum, 1992; Ragan, 1992) which is the most commonly used one in supertree studies and was also used in the formal definitions of the MRF method (Chen et al. 2003; Burleigh et al. 2004; Eulenstein et al. 2004). MRF is based on the notion of *flip distance* from a character matrix *M* to a tree *T* (Chen et al. 2003; Eulenstein et al. 2004). This quantity equals the smallest number of 1 → 0 and 0 → 1changes (*flips*) that must be made to *M* so that the 1-set of each character of *M* corresponds to some cluster in *T*. An *MRF supertree* for *M* is a tree *T* that has minimum flip distance to *T*. We now define the above notions precisely.

Let *T* be a phylogeny over some subset of *S*, *v* be a node of *T*, and *M* be a character matrix for *S*. Let *z_j_*(*v*) denote the number of taxa that are in the 0*-*set of character *j* and also in the cluster at *v*; that is, *z_j_*(*v*) = |*Z_j_*∩ℒ(*T_v_*)|. Similarly, let *o_j_*(*v*) denote the number of taxa that are in the 1-set of character *j* as well as in the cluster at *v*; that is, *o_j_*(*v*) = |*O_j_*∩ℒ(*T_v_*)|. The *flip distance* of character *j* to *v* is defined as

(1) $$f_{j}(v)=z_{j}(v)+(|O_{j}|-o_{j}(v)).$$

Note that *f_j_* (*v*) is the number of changes needed to make character *j* correspond to the cluster at node *v*. The first term in the right hand side of the above equation is the number of 0 → 1 changes and the second term equals the number of 1 → 0 changes.

The flip distance of character *j* to *T* is

(2) $$f_{j}(T)=\underset{v\in T}{\text{min}}f_{j}(v)$$

The flip distance of character matrix *M* to *T* is

(3) $$f_{M}(T)=\sum_{j=1}^{m}f_{j}(T)$$

The flip distance of a profile τ to *T i*s

(4) $$f_{\tau }(T)=f_{M}(T),$$

where *M* is some matrix representation of τ. Note that *f*_τ_ (*T*) is well-defined, since all matrix representations of τ are column permutations of each other.

The *minimum-flip problem* is: Given a character matrix *M* over *S*, find a phylogeny *T* over *S* such that *f_M_* (*T*) is minimum. The *fixed-tree minimum flip problem* is: Given a character matrix *M* for *S* and a phylogeny *T* for *S*, compute *f_M_* (*T*).

## Heuristics for MRF

The MRF supertree problem is defined only for rooted trees (Chen et al. 2003; Burleigh et al. 2004; Eulenstein et al. 2004), and the rooting of a tree can affect its flip distance from a character matrix. Thus, unlike MRP, MRF supertree heuristics cannot use existing unrooted tree search algorithms. The details of the original MRF heuristic algorithm were not described by Eulenstein et al. (2004), which has led to some apparent confusion in critiques of MRF (e.g. Goloboff, 2005). Therefore, we fully describe the accelerated MRF heuristic.

Like its predecessor, the new MRF heuristic uses a hill climbing strategy that is similar to the one used for unrooted tree searches in PAUP* (Swofford, 2002). The initial tree is obtained through greedy taxon addition using a randomly-chosen order (in practice, several initial trees are usually generated). After the initial tree is obtained, the search proceeds iteratively. At each step it locates the best tree (the tree with the lowest flip distance) that can be obtained from the current tree by a *branch swap*. Each tree that can be generated by a single branch swap is called a *neighbor* of the current tree. If no neighbor has a lower flip distance, the search stops and the current tree is returned as the estimate of a MRF supertree. Otherwise, the current tree is replaced by its best neighbor. The improved run times reported here, compared to the run times in the previous MRF heuristic, are due to changes in the implementation of the branch swapping operations.

We consider three rooted branch swapping operations.

**Rooted Nearest Neighbor Interchange (rNNI)** Choose an internal node *v* of *T* and swap one of *v*’s children with *v*’s sibling. Note that *T* has 2*n* − 4 rNNI neighbors.
**Rooted Subtree Pruning and Regrafting (rSPR)** (See also Hein, 1990; Bordewich and Semple, 2004.) Choose a non-root node *v* of *T*, called a *prune node*. Prune the subtree *T_v_* by removing the edge between *v an*d its parent, suppressing the remaining degree-two node. Next, *regraft T_v_* into *T* − *T_v_* *a*s follows: Pick a node *u*, called the *regrafting node*, in *T* − *T_v_*. If *u* is the root, create a new root *p* and make *p* the parent of *u* and *v*. Otherwise, create a new vertex *p* that subdivides the edge between *u* and its parent, and make *p* the parent of *v*. Note that *T* has Θ(*n*^2^) rSPR neighbors.
**Rooted Tree Bisection and Reconnection (rTBR)** This operation extends rSPR by allowing the pruned subtree *T*′ = *T_v_* to be *re-rooted* before regrafting. Re-rooting is done as follows: (i) Suppress the root node of *T*′. (ii) Create a new root node *r* by subdividing an edge {*x, y*} in *T*′ into the edges {*x, r*} and {*y, r*}. We refer to this operation as *bending* edge {*x, y*}. Note that *T* has Θ(*n*^3^) rTBR neighbors.

The earlier MRF heuristic found the best neighbor by computing the flip distance of each such neighbor from scratch (Eulenstein et al. 2004). This failed to exploit the similarities between the current tree and its neighbors, and, consequently, was quite slow. The running times to find an optimal neighbor tree of a given *n*-taxon tree for rNNI, rSPR, and rTBR were *O*(*n^2^m*), *O*(*n^3^m*), and *O*(*n*^4^*m*), respectively. The new algorithms reduce these times by a factor of *n*, giving execution times of *O*(*nm*), *O*(*n*^2^*m*), and *O*(*n*^3^*m*), respectively. In all three cases, the key is to preprocess the tree to allow evaluation of the flip distance of each neighbor in *O*(1) time per character. Our procedures share some ideas with recently described parsimony heuristics (Ganapathy et al. 2003).

In the remainder of this section, we first describe a *bottom-up assignment* algorithm that is used in all our branch swapping procedures. We then describe the new rSPR and rTBR algorithms. Finally, we explain the implementation of greedy taxon addition, which relies on rSPR. We have experimentally determined that rNNI tends to produce trees with higher flip distances than rSPR and rTBR; nevertheless, for completeness, we describe the rNNI algorithm in the Appendix. Since the flip distance *f_M_* (*T*) can be obtained by computing *f_j_*(*T*) independently for each character *j* and adding up the results (see Equation (3)), the descriptions of all the algorithms to follow focus on the computation of *f_j_*(*T*) for a single character *j*.

### Bottom-up assignment

The algorithm traverses the input tree *T* in postorder, computing four quantities for each node *v: z_j_*(*v*), *o_j_*(*v*), *f_j_*(*v*), and *f_j_*(*T_v_*). Before the traversal starts, it computes the values of |*O_j_*| for every character *j*; this takes *O*(*nm*) time.

Consider a node *v* of *T*. If *v* is a leaf, we can easily compute *z_j_*(*v*), *o_j_*(*v*), *f_j_*(*v*), and *f_j_*(*T_v_*) in *O*(1) time. Now, suppose *v* is an internal node with children *u* and *w*, such that *z_j_*(*x*), *o_j_*(*x*), *f_j_*(*x*), and *f_j_*(*T_x_*) are known for *x* = *u, w*. Obviously,

(5) $$\begin{array}{c} z_{j}(v)=z_{j}(u)+z_{j}(w)\\ \text{and}\\ o_{j}(v)=o_{j}(u)+o_{j}(w). \end{array}$$

Given *z_j_*(*v*)*, o_j_*(*v*), and |*O_j_*|, the value of *f_j_*(*v*) follows from Equation (1); *f_j_* (*T_v_*) is given by

(6) $$f_{j}(T_{v})=\text{min}\{f_{j}(T_{u}),f_{j}(T_{w}),f_{j}(v)\}.$$

Thus, *z_j_*(*v*)*, o_j_*(*v*)*, f_j_*(*v*), and *f_j_*(*T_v_* ) can be obtained in *O*(1) time. Since there are 2*n* − 1 nodes in *T,* computing the four required values for every node of *T* takes time *O*(*n*) per character, for a total of *O*(*nm*) time.

When the bottom-up assignment is finished, *f_j_*(*T*) = *f_j_*(*T_v_*), where *v* is the root node of *T*, and *f_M_*(*T*) can be computed in *O*(*m*) time via Equation (3).

### Finding the best rSPR neighbor

The algorithm considers all possible prune nodes; for each such node, it computes the optimum regrafting node. A prune node *v* is processed in two steps. First, apply the bottom-up assignment algorithm to *T_v_* and *T* − *T_v_*. Thus, for each node *w* of each tree and each character *j* we have *z_j_*(*w*), *o_j_*(*w*), *f_j_*(*w*)*,* and *f_j_*(*T_w_*). Second, traverse the nodes of *T* − *T_v_* in preorder. Let the *k-*th node in the preorder sequence be *u_k_*; thus, *u*_1_ is the root of *T* − *T_v_*. At step *k*, we compute the flip distance of the tree *T*^(^*^k^*^)^ obtained by regrafting *T_v_* at *u_k_*. We now explain how to obtain *f_j_*(*T*^(1)^) in *O*(1) time and how to compute *f_j_*(T^(^*^k^*^)^)*, k* > 1, in *O*(1) time using the information computed for *T*^(^*^k^* ^− 1)^.

Let *p_k_* denote the parent of *v* and *u_k_* in the *k-*th rSPR neighbor tree. Let *r_j_*^(^*^k^*^)^ denote *f_j_*(*T*^(^*^k^*^)^ − *T_pk_*^(^*^k^*^)^). Define *r_j_*^(1)^ = +∞.

**Figure 1:**
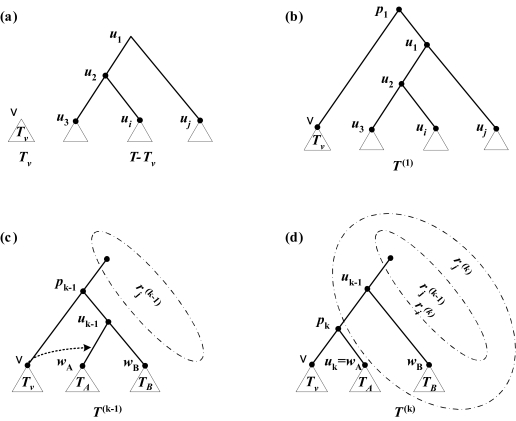
(**a**) The trees *T_v_* and *T* − *T_v_* obtained after a cut at node *v* (**b**) The first rSPR neighbor tree *T*^(1)^ obtained by regrafting at the root. (**c–d**) The transformation from *T*^(^*^k^* ^− 1)^ to *T*^(^*^k^*^)^.

Note that *p*_1_ is the root of the first rSPR neighbor tree (Figure 1(a–b)) and that

(7) $$\begin{array}{l} f_{j}(T^{\left(1\right)})=f_{j}\left(T_{p_{1}}^{\left(1\right)}\right)\\ =\text{min}\left\{f_{j}(p_{1}),f_{j}\left(T_{v}^{\left(1\right)}\right),f_{j}\left(T_{u_{1}}^{\left(1\right)}\right)\right\} \end{array}$$

The value of *f_j_* (*p*_1_) can be computed in *O*(1) time using Equations (1) and (5) and the information stored at the roots of *T_v_* and *T* − *T_v_*. Note that *f_j_* (*T_v_*^(1)^) equals *f_j_* (*T_v_*), which is known, and *f_j_* (*T_u_*__1__^(1)^) equals *f_j_* ((*T* − *T_v_*)*_u_*__1__), which is also known. Thus, *f_j_* (*T*^(1)^) can be obtained in *O*(1) time.

Assume that *T*^(^*^k^* ^− 1)^, *k* > 1, has been processed. We now describe how to process *T*^(^*^k^*^)^ (Figure 1 (c–d)). Let *w_A_* and *w_B_* denote the left and right children of *u_k_* _− 1_in *T*^(^*^k^* ^− 1)^ and let *T_A_* = *T_w_*_*_A_*_^(^*^k^* ^− 1)^ and *T_B_* = *T_w_*_*_B_*_^(^*^k^* ^− 1)^. Without loss of generality, we assume that *u_k_* = *w_A_*.

Assume that we know *r_j_* ^(^*^k^* ^− 1)^. For *T*^(^*^k^*^)^ we have

(8) $$r_{j}^{\left(k\right)}=\text{min}\{r_{j}^{\left(k-1\right)},f_{j}(u_{k-1}),f_{j}(T_{B})\}.$$

Since, the cluster at *u_k_* _− 1_ in *T*^(^*^k^*^)^ is the same as the cluster at *p_k_* _−1_ in *T*^(^*^k^* ^− 1)^, the above expression can be evaluated in *O*(1) time. Now,

(9) $$f_{j}(T^{\left(k\right)})=\text{min}\left\{r_{j}^{\left(k\right)},f_{j}\left(T_{p_{k}}^{\left(k\right)}\right)\right\}.$$

Note that *f_j_* (*T_p_*_*_k_*_^(^*^k^*^)^) = min {*f_i_*(*p_k_*), *f_j_*(*T_v_*), *f_j_*(*T_A_*)}, so *f_j_* (*Tp*_*_k_*_^(^*^k^*^)^) can be computed in *O*(1) time given the information available at *T_v_* and *T_A_* from the preprocessing step. Hence, *f_j_* (*T*^(^*^k^*^)^)can be obtained from *T*^(^*^k^* ^− 1)^ in *O*(1) time using Equation (9). Thus, the best regrafting node for *T_v_* can be found in *O*(*n*) time per character. Since there are *O*(*n*) choices for *v*, this leads to a time of *O*(*n*^2^) per character, and *O*(*n*^2^*m*) total, to find the best rSPR neighbor of *T.*

### Finding the best rTBR neighbor

rTBR differs from rSPR in that it may re-root *T_v_* before attaching it to *T* − *T_v_*. To handle rerooting efficiently, we use a *three-way assignment* approach, similar to the one used for parsimony by Ganapathy et al. (2003). We now outline the main ideas of this method.

**Figure 2:**
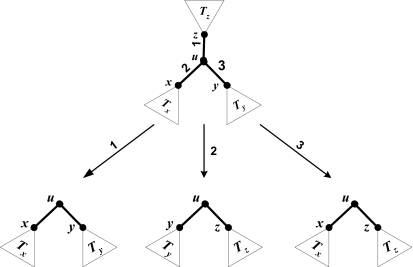
Internal node *u* and the three possible pairs of subtrees it may have, depending on the rooting. Each requires a different assignment.

Consider an internal node *u* as shown in Figure 2. The subtrees of *u* change, depending on whether the new root is in the direction of edges 1, 2, or 3; the subtrees of *u* are {*T_x_,T_y_*}, {*T_y_,T_z_*}, or {*T_x_,T_z_*}, respectively. A *three-way assignment* is a labeling of each vertex *u* with three lists of values 〈*z_j_*(*u*)*,o_j_*(*u*)*, f_j_*(*u*)*, f_j_*(*T_u_*)〉, one for each possible rooting. Such an assignment can be obtained in *O*(*n*) time per character by doing a bottomup assignment to find the assignments for the first rooting, and then doing a topdown preorder traversal to update the assignments for the other two rootings.

After computing a three-way assignment for the pruned subtree *T_v_*, we have, for every possible re-rooting *t* of *T_v_*, the information needed to find the best possible regrafting of *t* into *T* − *T_v_*, using the method earlier described for rSPR. This takes *O*(*n*) time per character for each fixed re-rooting *t*. Since there are *O*(*n*) possible re-rootings of *T_v_* and *O*(*n*) choices for *v*, the time required to find the best rTBR neighbor is *O(n*^3^*)* per character and *O*(*n*^3^*m*) total.

### Greedy taxon addition

The greedy search begins with a unique initial tree formed from the first two taxa in the input data set. The third taxon is inserted into every possible branch of the initial tree to form all possible three-taxon trees. The three-taxon tree with minimum flip distance is chosen. Each successive taxon is added in this way until a complete tree is obtained.

To find the best place to add the *k-*th taxon to the (*k* − 1)-taxon tree, we use our optimum rSPR neighbor algorithm. In this case, the tree being grafted has a single node containing taxon *k* and there are 2*k* − 3 ways to add this taxon to the (*k* − 1) -taxon tree. The time per regraft is *O*(*km*), yielding a total running time of *O*(*n^2^m*) for the entire addition sequence. We note that the performance the MRF heuristic may be improved by repeating the greedy taxon addition using different permutations of the taxa and thus generating multiple starting trees.

## Data Sets and Results

We examined the performance of MRF, and compared it to two other supertree methods, MRP and MMC, using three large, empirical data sets. MRF supertree analyses, implemented in HeuristicMFT2 (Chen, 2005), used rSPR and rTBR branch swapping on three random addition sequence replicates and saved a maximum of ten trees. MRP supertrees were constructed using PAUP* (Swofford, 2002), and used TBR branch swapping on three random-addition sequence replicates and saved a maximum of 100 trees. MMC supertrees were constructed with a program supplied by Rod Page (Page, 2003). The data sets (see Table 1) were taken from large, published supertree studies of marsupials (Cardillo et al. 2004), mammals (Price et al. 2005), and legumes (Wojciechowski et al. 2000).

The performance of each supertree method was evaluated by measuring the degree to which the supertrees agree with the input trees (e.g. Eulenstein et al. 2004). Two measures were used for this purpose: 1) the average MAST-fit between the supertree and the input trees and 2) the average triplet-fit from the supertree to the input trees. The MAST-fit between a supertree and an input tree is the number of leaves in their maximum agreement subtree (Gordon, 1980; Kubicka et al. 1992) divided by the number of leaves in common between the two trees. This was calculated using PAUP* (Swofford, 2002). The triplet-fit from a supertree to an input tree is 1 − (*d* + *r*)/(*d* + *r* + *s*), where *s* is the number of rooted triplets that are identically resolved in the supertree and the input trees, *d* is the number of triplets resolved differently in both trees, and *r* is the number of triplets resolved in the input tree but not in the supertree (Page, 2002). The triplet-fit, unlike the MAST-fit, is an asymmetric similarity measure. If there was more than one optimal supertree, we present the average score of all optimal supertrees to each of the input trees. In addition to measuring the quality of the supertrees, we also compared the CPU-time for each supertree method to provide a rough estimate of the computational requirements for each method. All the analyses were conducted on a Linux platform with a 3.0 GHz Pentium IV processor.

In the analyses of each of the three data sets, the MRF supertree had an equal or higher average MAST-fit and triplet-fit to the input trees than MMC or MRP supertrees (Table 2). In the marsupial and Cetartiodactyla data sets, the MAST and triplet-fit scores for the MRP supertrees were very similar to the scores for the MRF supertrees, while the scores for the MMC supertree were lower (Table 2). However, in the supertree analyses of the legume data set, the MAST and triplet-fit scores of the MRF supertree were noticeably (≥6%) higher than for the MMC or MRP supertrees. Also in the analyses of the legume data set, the triplet-fit score for the MRP supertree was higher than that of the MMC supertree, but the MAST-fit score of the MMC supertree was higher than that of the MRP supertree (Table 2). There was little if any difference in the performance of rSPR or rTBR algorithms in MRF analyses, though rTBR analyses required more CPU time than rSPR analyses (Table 2). In the analyses of the marsupial and Cetartiodactyla data sets, MRF with rSPR still required the most CPU time of the three supertree methods, but in the analysis of the legume data set, MRF with rSPR branch swapping used less CPU time than the MRP heuristic (Table 2).

## Discussion

The new heuristic algorithm makes MRF analyses feasible for large empirical data sets. The previous MRF algorithm was not only slow, its performance and implementation were questioned (Goloboff, 2005). Eulenstein et al. (2004) reported that the previous rSPR heuristic performed better than the rTBR heuristic for MRF. Goloboff (2005, p 289) interpreted this to mean that “SPR usually produced a better agreement with the model [true] tree.” However, Eulenstein et al.’s (2004) statement only referred to an anecdotal observation that rSPR was faster than rTBR and that the flip distances of rSPR and rTNR trees were very similar if not identical. In this study, we again observed that the MRF heuristic with rSPR branch swapping is much faster than the heuristic with rTBR branch swapping, and that both algorithms yield trees with similar flip distances (Table 2). The similarity between the performance of rSPR and rTBR may seem intuitively surprising (e.g. Goloboff, 2005), but it likely results from the nature of rooted branch swapping. rSPR and rTBR differ in that the latter may reroot the pruned subtree before regrafting. In most situations, it appears that rerooting does not reduce the flip distance. That is, the best rSPR and rTBR neighbors usually have the same flip distance. The implementation of the new heuristic also fixes a bug in the implementation of the previous MRF heuristic that caused the program to save suboptimal trees with rTBR and rNNI branch swapping (see Goloboff, 2005). Both rSPR and rTBR heuristics generally produce supertrees with lower flip distances than supertrees produced with rNNI heuristics (not shown). Though there appears to be little benefit in using the rTBR as opposed to rSPR heuristic in a quick MRF analysis, a thorough MRF analysis should include rTBR branch swapping.

The speed of the new heuristic makes it possible to assess the performance of the MRF supertree method using data sets that would have been too computationally demanding for the previous heuristic method. In fact, these are among the first reported MRF analyses using empirical data sets (but see Burleigh et al. 2004). In all three analyses, MRF appears to perform at least as well and often better than MMC and MRP (Table 2). The results also emphasize the differences that may exist between MRP and MRF supertrees. In previous simulation and empirical studies that used small input trees, the average similarity scores of MRP and MRF supertrees to the input trees were nearly identical (Chen et al. 2003; Burleigh et al. 2004; Eulenstein et al. 2004). However, this does not necessarily mean that MRF and MRP supertrees are similar to each other. In the analyses of the marsupial and Cetartiodactyla data sets, there is a notable difference in flip distance and parsimony scores of the MRF and MRP supertrees, even though the similarity of MRF and MRP supertrees to the input trees appears nearly identical. In the analysis of the legume data set, the difference in the parsimony scores and flip distances of the MRF and MRP supertrees is much larger (Table 2). These examples also demonstrate that the parsimony score of a supertree based on its binary matrix representation may not be a good indicator of the similarity of the supertree to the input trees. MRF trees with higher (worse) parsimony scores resemble the input trees more than the optimal MRP trees (Table 2). In these cases, minimizing the flip distance appears to be a better optimality criterion than minimizing the parsimony score. The legume supertree analyses also demonstrate that the MMC supertree method, which uses no true optimality criterion, can produce supertrees that appear more similar to the input trees than MRP. Thus, it may be unwise to rely solely on an MRP supertree analysis.

A good supertree method must balance computational speed with accuracy. For example, the MMC supertree method has a fast polynomial time algorithm (Page, 2002), but it often results in low quality supertrees (Table 2; Eulenstein et al. 2004). Conversely, the MRF supertree method appears to be accurate relative to other supertree methods, but previously its heuristics were too slow for large supertree studies (Eulenstein et al. 2004). However, the availability of heuristics should not dictate one’s choice of supertree methods. Rather, the properties of a supertree method should motivate the development of useful heuristics. Though a number of supertree methods have been proposed (see Bininda-Emonds, 2004), there has been much less focus on developing fast implementations of these methods. This study demonstrates that such work can benefit supertree analyses. We do not suggest that MRF is now the optimal supertree method. In some cases, MRF may exhibit undesirable properties (e.g. Goloboff, 2005; Wilkinson et al. 2005), and the speed of the new heuristics may still be a limitation for building supertrees with many thousand taxa or for implementing supertree bootstrapping replicates (e.g. Creevey et al. 2004; Philip et al. 2005; Burleigh et al. 2006). Still, with the new heuristics, MRF is, in many cases, a viable supertree method that should be considered along with other methods.

## Figures and Tables

**Figure 3:**
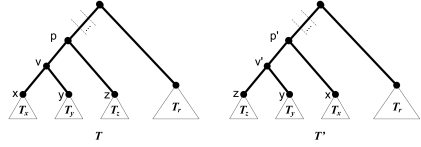
A rNNI operation on an internal node v of T.

**Table 1:** Supertree data sets. The second column lists the total number of input trees in each data set, and the third column lists the number of taxa that are found in the set of all input trees. The last column lists the number of characters in the binary matrix representation of the set of input trees.

Data set	Num. of input trees	Num. of taxa	Num. of characters
Marsupial	158	267	1775
Cetartiodactyla	201	290	1975
Legume	20	571	765

**Table 2:** Results of the supertree analyses of three empirical data sets. The triplet-fit and MAST-fit columns show the average triplet-fit or MAST-fit distances of the input trees to the supertree. The Pars. score column shows the parsimony score of the supertree based on the binary matrix representation of input trees, and the Flip dist. column shows the minimum flip distance of the supertree based on the binary matrix representation of input trees. CPU time is the computational time for each supertree algorithm.

Data set	Supertree	Triplet-fit	MAST-fit	Pars. score	Flip dist.	CPU time (sec)
Marsupial	MMC	0.544	0.542	3891	3058	164
	MRP	0.823	0.713	2274	823	583
	MRF(rSPR)	0.823	0.717	2296	801	989
	MRF(rTBR)	0.823	0.717	2594	801	1398
Cetartiodactyla	MMC	0.489	0.508	5017	4339	144
	MRP	0.796	0.654	2510	904	805
	MRF(rSPR)	0.803	0.659	2524	893	2258
	MRF(rTBR)	0.804	0.659	2523	893	2895
Legume	MMC	0.713	0.711	1489	1567	39
	MRP	0.789	0.663	962	710	6884
	MRF(rSPR)	0.849	0.764	1043	397	4958
	MRF(rTBR)	0.856	0.764	1041	392	8099

## Appendix

### Appendix: Finding the Best rNNI Neighbor

A rNNI operation on an internal node *v* of *T* resulting in a neighbor tree *T*′ is depicted in Figure 3. Observe that *T* and *T*′ have the same clusters, except that *T*′ does not contain cluster ℒ(*T**_v_*) = ℒ (*T**_x_*)∪ ℒ (*T**_y_*) and that *T*′ has a cluster ℒ(*T*′*_v_*_′_) = ℒ (*T*′*_z_*) ∪ ℒ(*T*′*_y_*) not present in *T*.

Prior to starting the search for the best rNNI neighbor, we execute the bottom-up assignment algorithm. Next, we traverse *T* in preorder, computing *f**_j_*(*T* − *T**_v_*) for each node *v* of *T* as follows. If *v* is the root of *T*, then *T* − *T**_v_* is an empty tree, denoted by ∅, and we define *f**_j_*(∅) *=* +∞. Now, suppose *v* has parent *p* and sibling *z* and assume that *f**_j_*(*T* − *T**_p_*) has been correctly computed. Then,

$$f_{j}(T-T_{v})=\text{min}\{f_{j}(T-T_{p}),f_{j}(p),f_{j}(T_{z})\}$$

Since *f**_j_*(*p*) and *f**_j_*(*T**_z_*) are known after bottom-up assignment, *f**_j_*(*T* − *T**_v_*) can be computed in constant time. There are 2*n* − 1 nodes in a binary tree *T*, and each visit of a node *v* takes constant time. Thus, the entire preorder traversal takes *O*(*n*) time per character, and *O*(*nm*) total.

Thus, we have *z**_j_*(*v*), *o**_j_*(*v*), *f**_j_*(*v*), and *f**_j_*(*T**_v_*), and *f**_j_*(*T* − *T**_v_*) for each node *v* and every character *j*. We now show how this information can be used to obtain the flip distance of each neighbor tree in constant time per character.

By Equation (2),

(10) $$f_{j}(T^{\prime })=\text{min}\{f_{j}({T^{\prime }}_{v}),f_{j}(T^{\prime }-{T^{\prime }}_{v})\}.$$

We now argue that *f**_j_*(*T*−*_v_*) and *f**_j_*(*T*′ − *T*′*_v_*) can be computed in *O*(1) time, and, thus, so can *f**_j_*(*T*′). Note that

(11) $$\begin{array}{l} f_{j}({T^{\prime }}_{v^{\prime }})=\text{min}\{f_{j}(v^{\prime }),f_{j}({T^{\prime }}_{z}),f_{j}({T^{\prime }}_{y})\}\\ =\text{min}\{f_{j}(v^{\prime }),f_{j}(T_{z}),f_{j}(T_{y})\}. \end{array}$$

The values of *f**_j_*(*T**_z_*) and *f**_j_*(*T**_y_*) are known, while *f**_j_*(*v*′) can be obtained in constant time using the precomputed values of *z**_j_* and *o**_j_* for nodes *z* and *y*, and Equations (1) and (5). Thus, *f**_j_*(*T*′*_v_*_′_) can be obtained in constant time.

On the other hand,

(12) $$\begin{array}{l} f_{j}(T^{\prime }-{T^{\prime }}_{v^{\prime }})=\text{min}\{f_{j}(T^{\prime }-{T^{\prime }}_{p}),f_{j}(p^{\prime }),f_{j}({T^{\prime }}_{x})\}\\ =\text{min}\{f_{j}(T-T_{p}),f_{j}(p^{\prime }),f_{j}(T_{x})\}. \end{array}$$

The values of *f**_j_*(*T* − *T**_p_*) and *f**_j_*(*T**_x_*) are known, while, like *f**_j_*(*v*′) above, *f**_j_*(*p*′) can be obtained in *O*(1) time from the pre-computed information.

Hence, for each rNNI neighbor *T*′ of *T*, *f**_j_*(*T*′) can be computed in *O*(1) time and *f**_M_*(*T*′) can be computed in *O*(*m*) time for a matrix of *m* characters. Thus, the best rNNI neighbor can be found in time *O*(*nm*).
